# Feasibility of a new ‘balanced binocular viewing’ treatment for unilateral amblyopia in children aged 3–8 years (BALANCE): results of a phase 2a randomised controlled feasibility trial

**DOI:** 10.1136/bmjopen-2023-082472

**Published:** 2024-07-30

**Authors:** Annegret Hella Dahlmann-Noor, John A Greenwood, Andrew Skilton, Daniel Baker, Mohamed Abbas, Emma Clay, Payal Khandelwal, Denise Dunham, Siobhan Ludden, Amanda Davis, Hakim-Moulay Dehbi, Steven C Dakin

**Affiliations:** 1 Institute of Ophthalmology, University College London, London, UK; 2 NIHR Moorfields Biomedical Research Centre, London, UK; 3 Moorfields Eye Hospital City Road Campus, London, UK; 4 Experimental Psychology, University College London, London, UK; 5 King's College London, London, UK; 6 Department of Psychology, University of York, York, UK; 7 Cambridgeshire Community Services NHS Trust, Saint Ives, UK; 8 University College London, London, UK; 9 School of Optometry, Auckland, New Zealand; 10 University of Auckland, Auckland, New Zealand

**Keywords:** ophthalmology, paediatrics, randomized controlled trial

## Abstract

**Objectives:**

This study aimed to evaluate the safety of dichoptic balanced binocular viewing (BBV) for amblyopia in children, plus feasibility, adherence, acceptability, trial methodology and clinical measures of visual function.

**Design:**

We carried out an observer-masked parallel-group phase 2a feasibility randomised controlled trial.

**Setting:**

Two study sites, a secondary/tertiary and a community site.

**Participants:**

We enrolled 32 children aged 3–8 years with unilateral amblyopia who had completed optical adaptation where indicated. 20 children attended the 16-week exit visit (retention 63%).

**Interventions:**

Children were randomised to BBV (movies customised to interocular acuity difference at baseline) for 1 hour a day (active intervention) or standard management as per parental choice (part-time occlusion or atropine blurring, control). All interventions were used at home, daily for 16 weeks.

**Primary outcome measure:**

‘VacMan suppression test’ of interocular balance at 16 weeks from randomisation. Secondary outcome measures: feasibility outcomes (recruitment and retention ratios, adherence with the allocated intervention); safety outcomes at other time points (changes in prevalence of diplopia, manifest strabismus, suppression/interocular balance on a range of tests); efficacy outcomes (clinical measures of visual function, such as best-corrected visual acuity, BCVA). Outcome measures were identical to those planned in the protocol.

**Results:**

Primary outcome: At baseline, values for the interocular balance point were higher (indicating greater suppression of the amblyopic eye) in the occlusion group than in the BBV group. These values shifted downwards on average for the occlusion group, significantly decreasing from baseline to week 16 (t_8_=4.49, p=0.002). Balance values did not change between baseline and week 16 for the BBV group (t_9_=−0.82, p=0.435). At 16 weeks, there was no statistical difference in interocular balance/suppression change over time between the two arms. The difference at follow-up between the arms, adjusted for baseline, was −0.02 (95% CI −0.28 to 0.23, p=0.87). Feasibility: We prescreened 144 records of potentially eligible children. Between 28 October 2019 and 31 July 2021, including an interruption due to the COVID-19 pandemic, 32 children were screened and randomised (recruitment rate 22%), 16 to BBV and 16 to standard treatment. 20 children attended the 16-week exit visit (retention 63%). Mean adherence with BBV as proportion of viewing time prescribed was 56.1% (SD36) at 8 and 57.9% (SD 30.2) at 16 weeks. Mean adherence with prescribed occlusion time was 90.1% (SD 19.7) at 8 and 59.2% (SD 24.8) at 16 weeks.

**Secondary safety/efficacy outcomes:**

One child in the BBV arm reported transient double vision, which resolved; two reported headaches, which led to withdrawal. BCVA improved from mean 0.47 (SD0.18) logMAR at randomisation to 0.26 (0.14) with standard treatment, and from 0.55 (0.28) to 0.32 (0.26) with BBV. Outcomes at 16 weeks did not differ between treatments.

**Participant experience:**

Families were generally positive about BBV, but families found both patching and BBV difficult to integrate into family routines.

**Conclusions:**

Recruitment rates indicate that a future phase 3 trial will require multiple sites or a longer enrolment period. Retention and adherence rates were lower than anticipated, which will influence future study designs. Dichoptic treatment may be equal to occlusion treatment in safety and efficacy; headaches may lead to discontinuation. Integration into family routines may constitute a barrier to implementation.

**Trial registration number:**

NCT03754153.

**STRENGTHS AND LIMITATIONS OF THIS STUDY**

- The COVID-19 lockdowns disrupted participant flow from enrolment to follow-up visits, ultimately resulting in a reduced sample size.
- Feasibility data on enrolment, retention and adherence rates delivered unexpected results, which will inform future study designs, that is, need for multiple research sites, a longer enrolment period, higher sample size to accommodate withdrawals and missing data.
- We collected broad and in-depth participant experiences with the novel intervention, revealing unexpected difficulties with implementing balanced binocular viewing at home.
- Giving parents/caregivers the choice of standard-of-care treatment, that is, occlusion or atropine, ensured a real-world comparator; however, during the COVID-19 lockdowns, occlusion treatment became standard of care, with atropine used as second-line treatment, which will inform future study designs.

## Introduction

 Amblyopia is the most common vision deficit in children, affecting around 3%.^1 2^ Standard treatment by part-time occlusion/patching of the better-seeing eye is limited by poor adherence.^3^ Dichoptic treatments, where the image viewed by the better-seeing eye is reduced in contrast, encourage balanced processing of the input from both eyes at the level of the visual cortex.^1 4^

Three types of dichoptic treatments have emerged. The first generation of treatments was a falling-block game, typically played while wearing anaglyph red/green glasses.^5^,^7^ For amblyopia treatment, this should be used every day for several months, though children failed to engage for the required duration of treatment, due to a lack of variety. A game developed specifically for children’s amblyopia treatment, DigRush, is more promising^8^,^10^ but still does not offer much variety. Contrast modification of movies and/or television shows emerged as a modality giving access to an abundance of material.^11^,^13^ Our previous work on balanced binocular viewing (BBV) involved children and parents in the development of a library of movies appropriate for children of different ages and covering entertainment and educational content.^11^

Early results with these dichoptic treatments have been promising, with visual outcomes comparable to those expected with standard occlusion treatment, but higher adherence.^11^ A concern that is commonly expressed in relation to amblyopia treatment is the risk of inducing diplopia, when treatments seek to reduce interocular suppression. To assess the safety of dichoptic treatments in this regard, we set up a feasibility trial to collect data on enrolment, retention, acceptability of the new treatment, including adherence, and trial methodology, as well as to explore the safety and efficacy of our treatment.

The primary objective for this trial was to evaluate the safety of BBV at 16 weeks from randomisation by exploring whether BBV causes a change in suppression/interocular balance, which may precede the development of double vision (diplopia).^14 15^ Changes to the strength of either binocular fusion or interocular suppression can alter the likelihood of diplopia.^16 17^ Diplopia can occur in normal individuals through variations in either of those factors,^16 17^ and amblyopia involves more extreme changes. Intractable, irreversible diplopia has to date never been observed in dichoptic treatment trials, though occasional, intermittent diplopia has been reported,^9 10^ both with dichoptic treatment and standard of care.^5^ A measure of interocular balance is the most appropriate outcome for a dichoptic amblyopia treatment trial, reflecting changes in visual function and associated changes in motor co-ordination.^18^ However, at present, no clinical test is considered sufficiently sensitive to measure subtle changes in interocular balance.^19^ We, therefore, opted to use a previously validated test developed by our team, the VacMan suppression test,^20^ which asks the participant to detect elements of varying contrast presented to each eye.^11^

Secondary objectives were those appropriate for a feasibility trial: recruitment and retention ratios, adherence with the allocated intervention, safety outcomes at other time points, such as changes in prevalence of diplopia, manifest strabismus, suppression/interocular balance on a range of tests and clinical measures of visual function, such as best-corrected visual acuity (BCVA).

## Methods

### Trial design

The trial was designed as an observer-masked parallel-group phase 2a feasibility randomised controlled trial at two sites, with 1:1 allocation ratio between the two interventions. There were no changes to the methodology after trial commencement.

### Participants

Participants were children aged 3–8 years with unilateral amblyopia. Case definition and full inclusion and exclusion criteria have been previously published^21^ and are listed in online supplemental table 1. Data were collected at two sites: a secondary/tertiary site (Moorfields Eye Hospital at City Road and at St George’s Hospital in London), and a community site (Cambridge Community Services Trust eye clinic in Bedford). Potentially eligible children were identified among new patients with strabismus and amblyopia. Glasses were prescribed as needed, and children were monitored for eligibility during the optical adaptation phase, if any. Families were approached about trial participation if an interocular difference in BCVA persisted on two consecutive clinic visits at least 8 weeks apart.

Children aged 5 years and older were randomised to paper-based and/or electronic information material provided as part of the TRECA trial (Trials Engagement in Children and Adolescents).^22 23^ Families of children younger than 5 years were given paper-based information material only. All information material included versions for parents and age-appropriate material for children. After answering all questions, the research orthoptist obtained written consent from parent/carer and verbal or written assent from the child.

### Interventions

**Figure 1:**
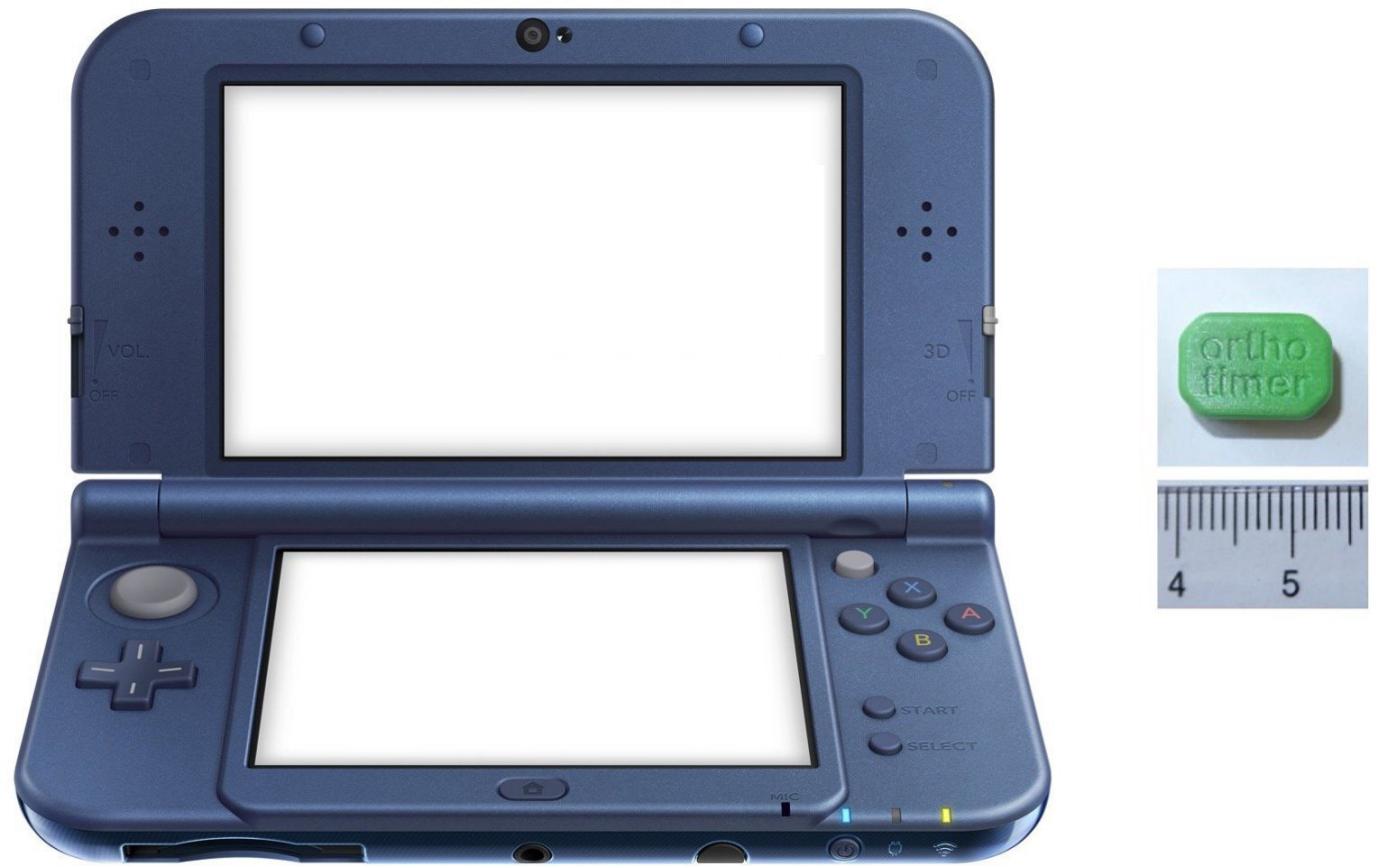
The handheld dichoptic games console used to administer the balanced binocular viewing treatment (left) and temperature sensor/logger with centimetre scale used as occlusion dose monitor for patching treatments (right).

Technical details of the active intervention have been published with the protocol.^21^ In brief, BBV consists of movies displayed on a handheld games console (Nintendo 3DS) with autostereographic display, which allows the delivery of different images to each eye without glasses, using a parallax barrier (figure 1). We customised commercial, age-appropriate movies for three levels of interocular acuity difference, presenting images of high, medium or low-level blur to the better-seeing eye. We asked children to view a movie for 60 min a day, either in one session or in two sessions of 30 min each.^21^ The control group received standard treatment, which is to offer parents/carers a choice between daily occlusion or twice weekly administration of atropine 1% eye-drops for pharmacological blurring of the better-seeing eye.^24 25^ The prescribed occlusion dose was 2 (moderate) or 6 hours a day (severe amblyopia).^26 27^ Parents/carers administered all interventions at home. We did not include a no-treatment control group, given both ethical and parental concerns about the effect of no-treatment or delayed-treatment conditions. Prior trials have nonetheless established the superiority of occlusion treatments over no-treatment for unilateral amblyopia.^28^ Instead, we focused here on the comparison between occlusion and BBV treatment approaches.

### Outcomes

#### Feasibility outcomes

Enrolment and retention were measured using relevant electronic log files (Microsoft Office Excel). For the experimental intervention, adherence data were collected by the device ‘usage’ function. Parents/carers of children using occlusion treatment were instructed on how to insert an occlusion dose monitor (Rollerwerk, Germany) between two layered standard eyepatches before placing it onto the child’s face (figure 1). Parents/carers using atropine eye-drops were asked to bring digital photographs of the child’s eyes to the study visits at 8 and 16 weeks. These photos were then reviewed by an unmasked observer who noted a total number of photos and the number which showed a dilated pupil. We calculated adherence as: (a) BBV: hours of usage as percentage of prescribed hours, and proportion of days on which BBV treatment was used; (b) occlusion: administered hours as percentage of prescribed hours; (c) atropine: photos showing enlarged pupil as percentage of total number of photos. To explore children’s and parents’/carers’ experience with BBV and trial methodology, the research orthoptist held a semistructured interview with the parent/carer at or after the study exit visit.

#### Primary outcome: safety

Our primary outcome was the change in interocular balance/suppression at 16 weeks relative to baseline. This measure derives from the VacMan suppression test, which asks the participant to indicate which of two elements appeared lighter, with elements of varying contrast presented to each eye (online supplemental figure 1).^11^ The luminance of the components was set using an interocular contrast ratio (R; 0–1), which determines the relative strength of the fellow and amblyopic eyes. Using QUEST,^29^ R was set to converge on the stimulus level that gave left/right reports with equal probability (ie, the point of interocular balance). R values above 0.5 indicate that a stronger signal is required in the amblyopic eye for that ghost to be reported as whiter (ie, suppression of the amblyopic eye), values close to 0.5 indicate balanced vision while those below 0.5 indicate that stronger signal is required in the fellow eye (suppression of the better-seeing eye).

#### Secondary outcomes: safety and efficacy

Further safety outcomes included questions about perception of double vision by the child, orthoptic assessment of eye alignment (manifest strabismus) and motor fusion, and tests of interocular suppression/balance (Sbisa bar,^30^ VacMan suppression test).^11^ As secondary efficacy outcomes, we recorded measures of visual function, that is, logMAR visual acuity and stereoacuity assessed using the Frisby stereotest and the VacMan random-pixel stereoacuity test.^11 31^

All of the above assessments were carried out at baseline and at 8 and 16 weeks from randomisation.

There were no changes to assessments or measurements after the pilot trial commenced. The protocol did not include prespecified criteria used to judge whether, or how, to proceed with a future definitive trial.

### Sample size

The target sample size was calculated on the visual acuity endpoint, critical to inform the design of the future phase 3 trial. A sample size of 44 evaluable patients was calculated to have 90% power with 5% two-sided alpha to detect a difference in the change of 0.225 logMAR in visual acuity between the two treatment arms, assuming an SD of the change of 0.22^11^ We expected a drop-out rate of 10%. The actual number of children enrolled was 32; enrolment was discontinued when funding had been exhausted. We did not carry out an interim analysis, and the protocol did not include stopping guidelines.

### Randomisation

#### Sequence generation

Children were randomised to either BBV or standard treatment with a 1:1 allocation ratio, stratified by parental choice of control treatment, level of interocular acuity difference and type of amblyopia, using minimisation with a random element to ensure that the researcher randomising the patient did not know what the next treatment allocation was going to be.

#### Allocation concealment mechanism

Both sites used the web-based Sealed Envelope system for sequence generation.

#### Implementation

Enrolment and assignment of the intervention were carried out by research orthoptists.

### Masking

It was not possible to mask children and parents/carers to the assigned intervention. Assessments were carried out by an orthoptist masked to the assigned intervention; we asked families not to disclose their allocated intervention to the masked orthoptist. In order to maintain masking in the atropine group, we asked families to discontinue treatment 2 weeks prior to the study visits at 8 and 16 weeks from baseline.

### Analytical methods

Patients’ baseline characteristics are summarised as mean±SD (or median±IQR in case of strong departure from normality) for continuous variables, and count+frequencies for categorical variables. The primary endpoint, as well as other endpoints that were continuous in nature, was analysed using analysis of covariance (ANCOVA). The regression models included the treatment variable and the baseline value of the endpoint of interest. The estimate from ANCOVA (referred to as the ‘treatment effect’) is the additional difference in the BBV intervention arm compared with the delta (change from baseline) in the occlusion/control arm. Examinations of the within-arm effectiveness of treatments were examined using paired-sample t-tests. A p<0.05 was considered statistically significant.

### Patient and public involvement

We had intended to involve up to six parents or carers of children with amblyopia, to form a parallel advisory group for the study recruited via Moorfields clinics. The role of the advisory group would have been to codesign participant information and consent materials for the study and a standardised set of questions for the semistructured interviews exploring families’ experiences of taking part, to provide ongoing advice on participant recruitment and retention, to provide a contextualisation of study findings from lived-experience, and to have coauthored appropriate formats for the dissemination of the findings to professional and non-professional audiences. At least one advisory group member would have also sat on the study steering group. The advisory group would have been made up of a new cohort of parents and carers who had not been involved in informing the initial feasibility and design of the study^19^ to allow for wider perspectives and more diverse experiences. All members of the advisory groups would have been reimbursed in line with standard NIHR involvement rates.

We were unable to recruit any interested parents or cares for the advisory group, therefore, we relied on the previous experience and expertise of the study team and the patient and public involvement team at NIHR Moorfields Biomedical Research Centre to inform the design of participant information, recruitment and retention strategies, and interview questions. After study initiation, through wider channels, we were able to recruit a carer representative to the steering group, who had experience with children of the appropriate age for the study, but who did not have specific experience with amblyopia. Due to the protracted course of research projects during the COVID-19 pandemic and in-hospital safety measures, we did lose contact with our steering group carer representative, and we were unable to conduct further attempts to recruit appropriate parent/carer representation.

Feedback to participants and dissemination were discussed at the meeting of the NIHR Young People’s Advisory Group for eye and vision research (eye-YPAG) on 6 July 2024, which was attended by 15 CYP aged 5–16 years. The group enquired about the target audience, pointing out that the children who took part in the BALANCE pilot trial are now 5 years older than at the start of the study, that is, 9–13 years. They felt that text summarising the trial and its findings would be too technical and boring and recommended a visual summary, that is, an infographic. The group reviewed a draft and recommended a simple structure, short paragraphs, bullet points, some small illustrations and a simple colour scheme. They also recommended not to include too many numbers or percentages and not to include graphs. The resulting infographic, which was then sent to participating families and posted on the participating institutions’ websites, is shown in online supplemental figure 1.

## Results

### Feasibility outcomes

#### Participant flow/recruitment rates

We enrolled and randomised 32 children (online supplemental figure 3—CONSORT (Consolidated Standards of Reporting Trials) flow chart).

144 children were identified as potentially eligible on prescreening of their clinical records during optical adaptation. We enrolled 32 participants (24%). Reasons for non-enrolment are shown in online supplemental table 2; the the most common were resolution of amblyopia during optical adaptation only (n=55), strabismus greater than 10 prism dioptres (n=7) and other reasons for not meeting eligibility criteria (n=25). Concerns relating to the COVID-19 pandemic played a role in 12 potential recruits (n=12).

#### Recruitment

The enrolment period was extended from 28 October 2019 to 31 July 2021, including an interruption due to the COVID-19 pandemic. The trial ended on 28 October 2021 with the last participant’s last visit.

#### Baseline data

Table 1 shows demographic and clinical characteristics at baseline.

**Table 1:** Demographic and clinical baseline characteristics, separately for the BBV and occlusion/control groups

	BBV	Occlusion
N	16	16
Girls; boys	10; 6	6;10
Study eye right; left	8; 8	8; 8
Mean age (SD) in years	4.81 (0.83)	5.13 (0.96)
Anisometropic amblyopia (n, %)	9 (56%)	8 (50%)
Strabismic amblyopia	4 (25%)	4 (25%)
Combined mechanism amblyopia	3 (19%)	4 (25%)
Mean (SD) spherical equivalent amblyopic eye in D	4.93 (3.06)	4.16 (3.21)
Mean (SD) spherical equivalent fellow eye in D	2.37 (2.83)	2.65 (2.75)
mean (SD) best-corrected visual acuity amblyopic eye in logMAR	0.55 (0.28)	0.47 (0.18)
Mean (SD) best-corrected visual acuity fellow eye in logMAR	0.06 (0.11)	0.09 (0.09)
mean interocular acuity difference (SD) in logMAR	0.49 (0.27)	0.38 (0.14)
Manifest strabismus with distance fixation on simultaneous and prism cover test (n, %)	4, 25%	5, 31%
Manifest strabismus with near fixation on simultaneous and prism cover test (n, %)	5, 31%	5, 31%

BBVbalanced binocular viewing

#### Numbers analysed/retention rates

11 children in the BBV and 9 in the control group used the allocated intervention and attended the 16-week exit visit. In the control group, eight used occlusion and one used atropine eye-drops. This means that 20 of 32 randomised participants (ie, 63%) completed the study (online supplemental figure 2: CONSORT flow diagram).

One child did not attend the week 16 visit but used the allocated treatment (occlusion) for the study period; however, exit data are not available for them. Six participants were withdrawn before the week 8 visit: three by the research team (two BBV, one occlusion), as the study was suspended during the COVID-19 lockdowns, and three by the family (one BBV, two occlusion). One family was not happy with the allocated intervention (occlusion), one child reported headaches while watching BBV movies (BBV) and one family did not attend the research visit and did not give a reason for withdrawing (occlusion). Three children were withdrawn at the week 8 visit, two for clinical reasons—one because of a risk of developing reverse amblyopia (occlusion), one because of an increase in manifest strabismus to greater than 10 prism dioptres since baseline visit (occlusion) and the third because of headaches (BBV) and parental request to change treatment. Two children were withdrawn between the weeks 8 and 16 visits by the family, as they were not able to use the allocated treatment as prescribed (one BBV, one occlusion).

#### Adherence

Occlusion dose monitor data were available for seven participants at 8 weeks and six at 16 weeks from randomisation. Mean adherence as proportion of occlusion time prescribed was 90.1% (SD 19.7) at 8 and 59.2% (SD 24.8) at 16 weeks. BBV adherence data were available for 11 participants at week 8 and 8 at week 16 from randomisation. Mean adherence as proportion of viewing time prescribed was 56.1% (SD36) at 8 and 57.9% (SD 30.2) at 16 weeks.

#### Unmasking

Unmasking of the orthoptist carrying out the study assessments occurred once, in the occlusion group. This family withdrew between weeks 8 and 16, as they were unable to administer the treatment and informed the masked orthoptist.

## Outcomes and estimation

### Primary outcome: safety

Change in interocular balance on VacMan Suppression test^11^ from randomisation to 16 weeks

**Figure 2:**
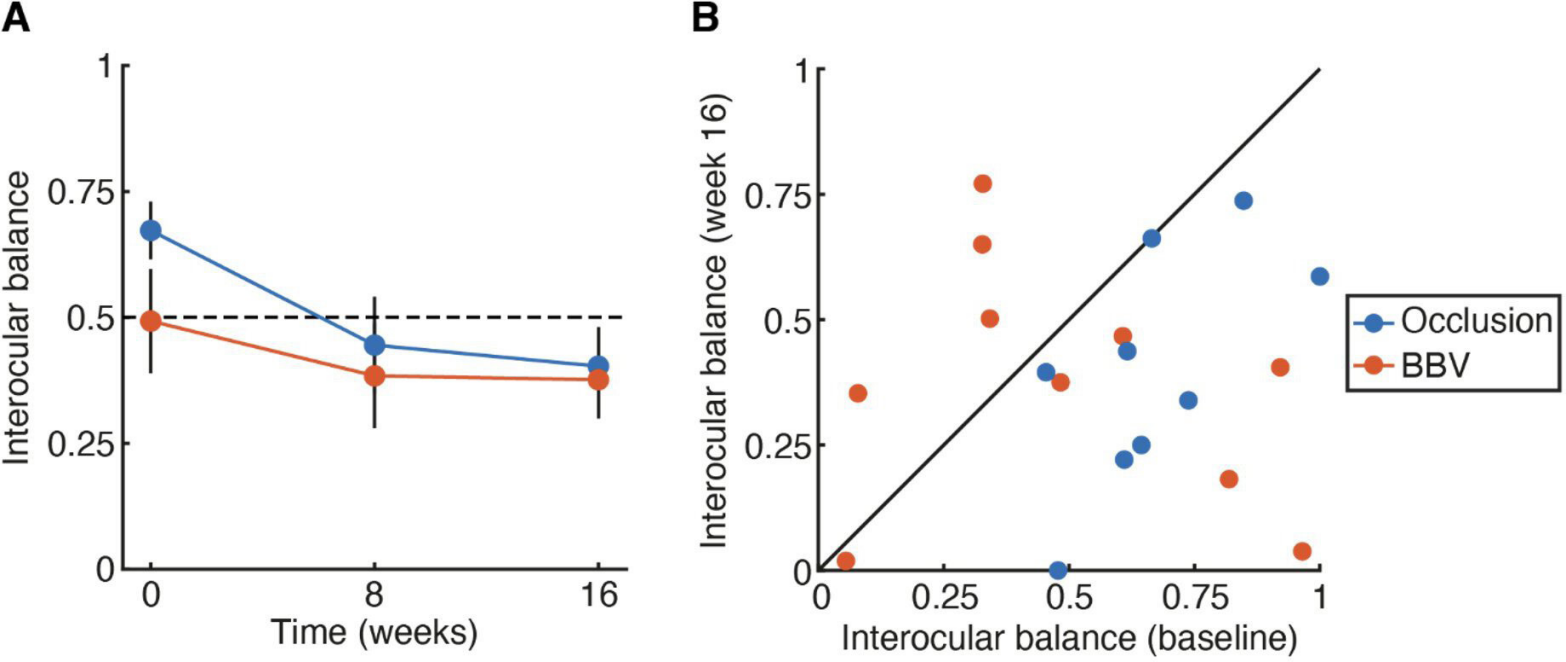
Interocular balance/suppres:sion values from the VacMan suppression test. (A) Thresholds at baseline, 8 and 16 weeks for each of the two treatment arms: occlusion (blue) and BBV (orange). Points show the mean; error bars the SEM values above 0.5 indicate suppression of the amblyopic eye, points below 0.5 indicate suppression of the fellow eye. (B) Interocular balance/suppression scores for individual children plotted at baseline (x-axis) compared with week 16 (y-axis).

The trajectory of interocular balance values from baseline to week 16 is presented in figure 2A, with individual values (comparing baseline and week 16) shown in figure 2B. Data are only presented for those with completed measures at all time points (n=9 for occlusion; n=10 for BBV). At baseline, values for the interocular balance point were higher (indicating greater suppression of the amblyopic eye) in the occlusion group than in the BBV group. These values shifted downwards on average for the occlusion group, significantly decreasing from baseline to week 16 (t_8_=4.49, p=0.002). Balance values did not change between baseline and week 16 for the BBV group (t_9_=−0.82, p=0.435), consistent with prior work. At 16 weeks, there was no statistical difference in interocular balance/suppression change over time between the two arms. The difference at follow-up between the arms, adjusted for baseline, is −0.02 (95% CI −0.28 to 0.23, p=0.87).

### Secondary outcomes: safety and efficacy

#### Safety/adverse events

Transient double vision was reported by one child in the BBV group during the first 8 weeks of treatment, which resolved and did not lead to withdrawal. No other cases were reported. Other adverse events leading to withdrawal were child-reported headaches, with two cases in the BBV group. Headaches were transient and resolved when treatment was stopped.

#### Efficacy outcomes: BCVA, Frisby stereoacuity, interocular suppression on Sbisa bar

**Figure 3:**
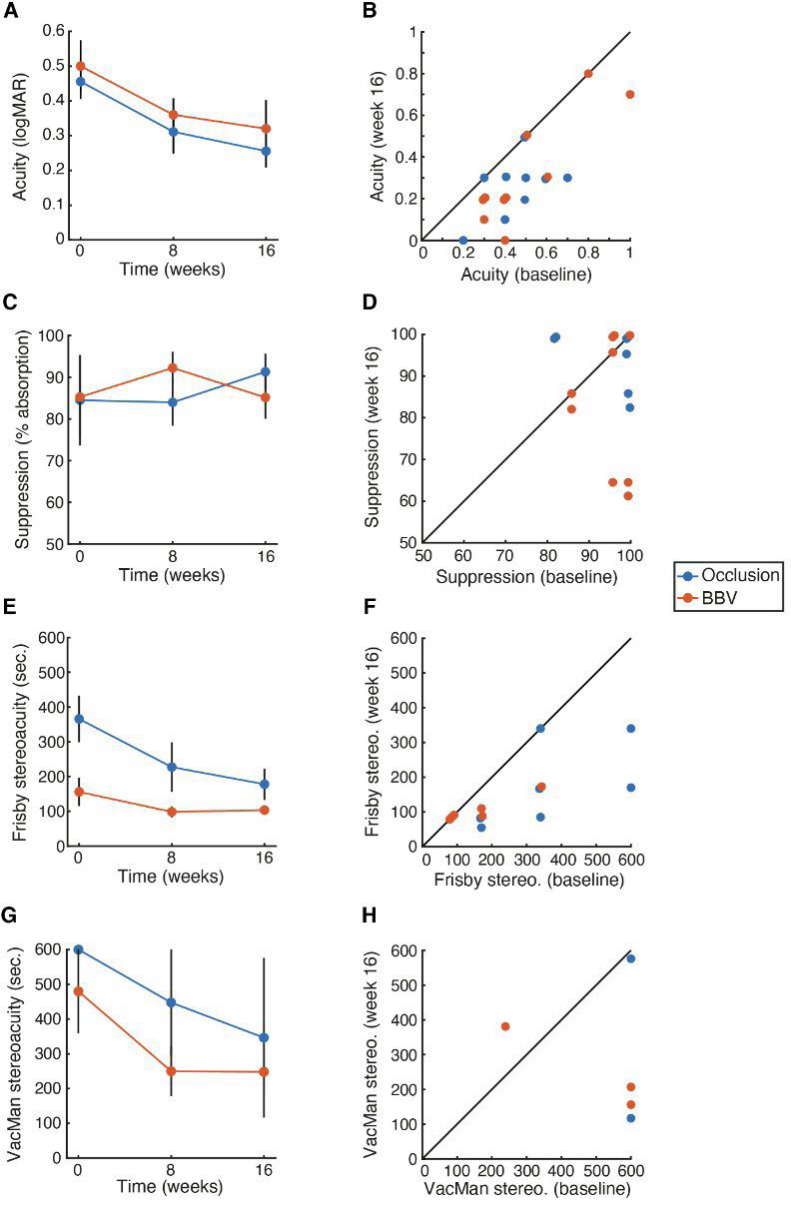
Secondary visual outcomes: (A) Mean values of best-corrected visual acuity (BCVA) in logMAR for the occlusion (blue) and BBV (orange) arms across the three time points. (B) Individual BCVA values at baseline (x-axis) and week 16 (y-axis). C. Mean Sbisa suppression values, plotted as in A. (D) Individual Sbisa values, plotted as in B. (E) Mean Frisby stereoacuity thresholds in seconds of arc. (F) Individual Frisby stereoacuity thresholds. (G) Mean VacMan stereoacuity thresholds in seconds of arc. (H) Individual VacMan stereoacuity thresholds. BBV, balanced binocular viewing.

Online supplemental table 3 and figure 3 report the secondary endpoints from a quantitative perspective. BCVA (using HOTV judgements) was poor for both groups and improved over the course of treatment (figure 3A). The improvement in BCVA from baseline to week 16 was significant for both the occlusion (t_8_=4.24, p=0.003) and BBV (t_9_=4.32, p=0.002) groups. Each gained the equivalent of two lines on a logMAR chart, though there was no significant difference between the two arms at 16 weeks (difference: 0.03, 95% CI −0.11 to 0.16, p=0.67). Improvements can be seen for almost all individuals, with comparison of baseline and week 16 in figure 3B.

Sbisa suppression (figure 3C for means and figure 3D for individuals) was high in both treatment arms to begin, and remained constant over the course of treatment for both groups. The difference between baseline to week 16 was non-significant for both the occlusion (t_8_=−0.53, p=0.612) and BBV (t_9_=0.01, p=0.993) groups. There was also no significant difference between the two arms at 16 weeks (difference −6.9, 95% CI −22.6 to 8.73, p=0.36).

Frisby stereoacuity values (figure 3E for means and figure 3F for individuals) could only be obtained at all three time points for seven children in the occlusion arm and six in BBV. Values were high at baseline, particularly for the occlusion treatment arm, and reduced over the course of treatment. The difference between baseline to week 16 was significant for occlusion (t_6_=3.51, p=0.013) but did not reach significance for BBV (t_5_=1.89, p=0.118) groups. Given the difference in baseline stereoacuity, it is difficult to determine whether this is due to the sample selection or differential efficacy. Nonetheless, there was no significant difference between the two arms at 16 weeks (difference: 16.5, 95% CI −77.7 to 110.8, p=0.70).

VacMan stereoacuity values (figure 3G for means and figure 3H for individuals) derive from judgements of random-pixel stereogram elements.^31^ Children found this particularly difficult, likely due to the pixel elements being too close to their acuity thresholds. Complete data at all time points was only obtained with two children in the occlusion arm and three with BBV. A trend towards improvements is nonetheless visible, with high values obtained at baseline, and a reduction by week 16 in both arms. These differences were, however, non-significant for both occlusion (t_1_=1.11, p=0.468) and BBV (t_2_=1.24, p=0.342) groups. Clear improvements are nonetheless evident in the majority of individuals (figure 3H), suggesting that larger samples may show clearer gains.

#### Participant experience

Table 2 summarises participant experience. Parents/carers were generally positive about attending research appointments; negative comments related to overall length of appointments. Overall, there seemed to be more positive comments about the BBV intervention compared with occlusion treatment; though interest in the device did decrease over time due to ‘limited choice of videos and older hardware’. Both arms had difficulties with integrating the intervention into the family routine. For occlusion treatment, this related to a dislike of drops or wearing a patch. In the BBV arm, this related to fitting in dedicated time to watching the device (eg, weekends and after school clubs) and competing with other distractions.

**Table 2:** Participant experience, separately for the BBV and control/occlusion groups

Participants	BBV	Occlusion
	n=7	n=6*
Age range (years)	5–7	4–7
Gender (female:male)	6:1	2:4
Study eye (right:left)	3:4	3:3
Theme 1: Child’s feelings about research appointments % (n)		
Positive	85.7 (6)	66.7 (4)
Negative	14.3 (1)	16.7 (1)
Neutral	14.3 (1)	7 (1)
Theme 2: Child’s feelings about intervention % (n)		
Positive	100.00 (7)	33.3 (2)
Lost interest	85.7 (6)	0.0 (0)
Negative	0.0 (0)	33.3 (2)
More positive over time	0.0 (0)	33.3 (2)
Neutral	0.0 (0)	16.7 (1)
Theme 3: Experience of integrating intervention into the family routine % (n)		
Able to fit it in	42.9 (3)	50.0 (3)
Difficult to fit it in	57.1 (4)	50.0 (3)
Theme 4: Reaction to intervention from friends/siblings/cousins % (n)		
Positive	28.6 (2)	33.3 (2)
Negative	14.3 (1)	0.0 (0)
No reaction reported	57.1 (4)	66.7 (4)
Theme 5: Changes in Child’s ability to take part in activities and pastimes % (n)		
Reported an improvement	100.0 (7)	83.3 (5)
No improvement reported	0.0 (0)	16.7 (1)

13 families were available for interview at the end of the study.

* One child received atropine, all others received patching treatment.

BBVbalanced binocular viewing

We did not find evidence that children on occlusion/patching treatment, which is more noticeable to peers, received more negative responses than those in the BBV device arm. There was one case of perceived jealousy from a sibling in the BBV group, but this resolved when they experienced the dichoptic display and found it ‘blurry and boring’. Consistent with the visual outcomes, all participants reported a subjective degree of improvement.

## Discussion

### Principal findings

The principal findings of this study concern enrolment and participant experience with dichoptic treatments. Enrolment may be slower than anticipated for future trials with similar inclusion criteria—particularly the requirement for completion of optical treatment prior to inclusion. In our study, this was the most common reason for non-enrolment, in 55 of 144 prescreened cases (38%), as the interocular difference in visual acuity often improves over time prior to enrolment to less than 0.2 logMAR. Future studies should be set up across multiple sites to shorten the enrolment phase. Child and parent experience with the novel approach revealed unexpected difficulties: parents found it difficult to integrate 1 hour of supervising the child during the movie session into the daily routine, children got bored of the movies on offer, despite a change of movies after 8 weeks, and two children reported headaches. During the COVID-19 pandemic lockdowns, amblyopia research visits were suspended, demonstrating that research into conditions that are not life-threatening or sight-threatening is vulnerable to external circumstances.

Our primary outcome measure was interocular suppression. Though suppression of the amblyopic eye reduced from baseline to week 16 in the patching group, there was no significant change in the BBV group. This is consistent with prior results showing improvements in visual outcomes with BBV in the absence of changes in suppression.^11^ Suppression values obtained with the VacMan suppression test were somewhat variable, with baseline levels that differed between the occlusion and BBV groups. This was not related to the method of measurement used: suppression values obtained with Sbisa filters showed a similar pattern, exhibiting neither significant change over the course of treatment, nor difference between treatments at week 16. The lack of change in suppression with BBV means that the risk of developing diplopia over the course of these treatments should be low, as we also observed in this study. Nonetheless, our secondary outcomes show clear improvements in vision following treatment. Visual acuity in the amblyopic eye improved to a similar extent to that of standard occlusion treatment, with children gaining two lines on a logMAR chart on average. Stereoacuity values also showed trends towards improvement, though the conclusiveness of these outcomes was limited by the small sample size and drop-out rates.

### Strengths and weaknesses of the study

The study suffered from enrolment difficulties, which were compounded by the COVID-19 lockdowns that disrupted every step of the participant flow: school vision screening programmes were halted, which meant that fewer children with sight problems were identified, fewer children started wearing glasses and fewer children became eligible to take part in the study. This reduction in eligible children continued for several months after restrictions were lifted. As a consequence, funding ended before the completion of the study. We reduced the sample size, as the main aim of this trial was to assess feasibility, and we felt that a smaller study would still permit conclusions about feasibility and inform the design of a future phase 3 trial.

A particular strength of this study is the wealth and depth of participant experience that we collected, which highlighted unexpected difficulties with novel dichoptic treatments such as BBV. This qualitative work in itself faced difficulties and limitations. Due to pandemic restrictions and a lack of parents’ interest to form an advisory group, questions were decided on by the study team. The survey did not undergo cognitive testing to ensure questions were universally understood and accessible to English speakers of other languages (ESOL). Several parents who responded to the survey were ESOL and did not request translation support; we cannot, therefore, be certain that all questions were universally understood. Questions were intended to be used for a written response survey to be completed by the parent with their child at the last study visit; however, due to pandemic restrictions, questions were instead delivered as a verbal interview over the phone, in some cases, several months after the last study engagement. Interviews were conducted by the study orthoptists rather than trained qualitative researchers and a summary of responses was recorded rather than a full transcript for analysis. As we carried out the survey by telephone, we only heard from the parent, but not directly from the child.

### Strengths and weaknesses in relation to other studies, discussing important differences in results

No other dichoptic treatment trial has published enrolment rates, including those designed as pilot studies.^7 8^ Similarly, no previous trial has explored how easy parents would find it to implement the new treatment at home, supervising their child for an hour a day. However, all trials have published adherence rates, collected either via parental diary or device-build usage data. For the first wave of dichoptic treatments, using a falling block game and anaglyph glasses, adherence has been reported as 22.2% using the treatment more than 75% of the prescribed time over 16 weeks,^5^ 64% using it more than 25% over 6 weeks^6^ and 83% using it more than 75% over 6 weeks^7^ ; the latter study also reported a mean use of 37 hours over 6 weeks (88% of prescribed).^7^ With the second type of dichoptic games, the Dig Rush game played again with anaglyph glasses, adherence was reported as 100% for the first 2 weeks, then falling to 82% for the next 2 weeks,^8^ 56% or 43% using the treatment more than 75% of the prescribed time over 8 weeks,^9 10^ and median 62% of prescribed usage time over 8 weeks.^10^ One study pointed out that games need to be age appropriate and varied to maintain interest and engagement.^32^

With the third type, contrast-modified movies, adherence rates increased 88.2% over 12 weeks, in a trial using VR headsets as displays, and which did not offer any treatment other than glasses to the control group and enrolled a substantial number of participants who had previously received other forms of amblyopia treatment, both of which are likely to have affected motivation.^12^ In a trial using Nintendo 3DS games consoles as a delivery platform, adherence of 95%–107% over 4 weeks was observed (some participants using the treatment more than prescribed), but longer-term data are not available.^12^

In our study, adherence data were only available for 40%–69% of participants per time point and treatment, limiting validity. In line with other occlusion treatment trials, we observed that mean adherence fell in the second 8-week interval, to 59% of the prescribed dose. Mean adherence in the BBV arm was around 56% at both time points, which is lower than reported in other studies.

Not all trials have reported efficacy figures in comparison to standard of care; those that did observed, as we did, an improvement in amblyopic-eye visual acuity similar to patching, both over short period of 2–6 weeks^12^ and over a clinically relevant time period of 12 or 16 weeks, and only in children younger than 7–8 years.^5^ When we designed this study, parents/carers were given a choice of occlusion or atropine as first-line treatments. This changed during the COVID-19 lockdowns, when monitoring for potential ‘reverse amblyopia’ with atropine was not possible; since then, occlusion treatment has become first-line treatment. In future trials, standard of care should be occlusion treatment, which would also reduce the risks linked with non-treatment for 2 weeks before study visit to maintain masking of atropine treatment. It was initially thought that dichoptic treatments may work faster,^8^ but more recent work did not confirm this finding.^12^ Our results are consistent with the latter observation.

Over the course of several dichoptic treatment trials, adverse event reporting for this new modality has become more standardised, and now includes four types of treatment-related adverse events: double vision, manifest strabismus, headaches and eyestrain. Of greatest concern to clinicians and regulatory authorities is permanent/intractable double vision. This event has to date never been observed in dichoptic treatment trials, though occasional, intermittent diplopia has been reported,^9 10^ both with dichoptic treatment and standard of care.^5^

The incidence of new onset of manifest strabismus or worsening of existing manifest strabismus by 10 prism dioptres or more tends to be similar to participants receiving standard of care^10^; one trial reported an incidence of new manifest strabismus in 6%,^13^ another new or worsening strabismus in 13% at 8 weeks from randomisation.^9^ Headaches have been reported in 8% of a trial of dichoptic movies viewed via VR headset.^13^ None were reported with movies viewed on a 3D games console, but this trial did not report any adverse events at all.^12^ It is, therefore, interesting to note that two of our participants reported headaches using the same delivery platform. One trial reported an increase in the frequency of headaches in 9%,^10^ an increase in eyestrain in 10% at 4 weeks and in 6% at 8 weeks for both.^10^ Another trial reported eyestrain in 3.6% of participants using dichoptic treatment.^6^

In our study, we used interocular balance, or suppression, as a safety outcome, as conventionally it is thought that reduction in suppression precedes the onset of double vision. We were reassured to observe that balance (suppression) remained, as it had in our previous work.^11^ Most dichoptic treatment trials did not measure suppression, probably because the relevant tests are not in clinical use. Those trials that did use a dichoptic global motion test observed early reduction in suppression at 2 weeks,^8^ minimal change at 6 weeks^6^ or no change at 2 and 6 weeks.^12^ Taken together, this appears to indicate that improvement in visual acuity may indeed not be associated with a change in suppression.

### Meaning of the study: possible explanations and implications for clinicians and policy-makers

As this study was not a full efficacy trial, we are not in a position to make recommendations about the role of novel dichoptic treatments in the management of amblyopia. However, our results on vision improvement match those observed in larger trials. It would, therefore, be appropriate to consider dichoptic treatments as a possible alternative to current treatment options, particularly for children with poor adherence to patching. Pending larger trials, dichoptic treatment could also be used in combination with patching treatment, that is, patching on school days and dichoptic viewing at weekends.

### Unanswered questions and future research

This study cannot give a definitive answer on safety and efficacy of dichoptic treatments for amblyopia; for this, a phase 3 randomised trial will be required. However, the participant experience detailed here indicates that before any such work is undertaken, research should systematically explore potential barriers to implementation of dichoptic treatments. Such research should use established frameworks such as the capability, opportunity and motivation of behaviour framework.^33^ This would allow the identification of critical determinants of adherence to this novel treatment, that is, capability to engage in movie-watching, social and environmental influences and emotional, cognitive or habitual responses to the new approach. In addition, the material available for viewing needs to be extended, to prevent the onset of boredom once the novelty of the new device-based treatment has worn off. Similarly, hardware platforms need to keep up with technological advances which children and families have become used to, for example use of smartphones as delivery devices.

Lack of time to attend research visits is a frequent barrier to enrolment. To mitigate this issue, future amblyopia treatment trials may wish to limit visits to the intervals used in standard clinical care. The length of study visits can also be a burden to participants, and investigators need to prioritise assessments essential to address the research question. On the other hand, it would be valuable to have data on the final degree of visual improvement and on potential regression once treatment is discontinued.

Patient and public involvement and engagement should also be more robust in future work and should include a YPAG such as Moorfields’ eye-YPAG as well as children with amblyopia and their parents, who can contribute their lived experience to the design of study protocols and materials, including appropriate surveys of the research experience. Inclusion of a behaviour change researcher and a trained qualitative researcher to carry out family interviews before and during the clinical evaluation would deliver high-quality insights within an acknowledged theoretical framework. With video-conferencing facilities now routinely used within the National Health Service (NHS), future studies should ensure that the voice of the child participating in the research is included, even when a face-to-face interview is not possible. Lastly, we would revisit the use of the Child Health Utility 9D index,^34^ which was ruled out during the initial PPI interviews undertaken at study design phase.^21^ Although not tailored to amblyopia treatment trials, it would allow the collection of health utility data using a standardised approach.

## supplementary material

10.1136/bmjopen-2023-082472 online supplemental file 1

10.1136/bmjopen-2023-082472 online supplemental file 2

10.1136/bmjopen-2023-082472 online supplemental file 3

10.1136/bmjopen-2023-082472 online supplemental file 4

10.1136/bmjopen-2023-082472 online supplemental file 5

10.1136/bmjopen-2023-082472 online supplemental file 6

10.1136/bmjopen-2023-082472 online supplemental file 7

## Data Availability

Data are available on reasonable request.
