# Supplementary material for: Feasibility of a new ‘balanced binocular viewing’ treatment for unilateral amblyopia in children aged 3–8 years (BALANCE): results of a phase 2a randomised controlled feasibility trial
Source: BMJ Open. 2024 Jul 30;14(7):e082472. doi: 10.1136/bmjopen-2023-082472 (PMC11407205; doi:10.1136/bmjopen-2023-082472)
Supplement: online supplemental file 1 [file bmjopen-14-7-s001.docx]

Supplementary Figure 1. (A) In the suppression task patients judged which of two “ghosts” – each made up of a mixture of dichoptically-presented light and dark ghosts and presented either side of VacMan - appeared to be lighter. For the depicted inter-ocular contrast ratio of 75%, the ghost presented to the amblyopic eye (AE) was a 0.75*L increment on one side (here the left) and a similar magnitude decrement on the other side, while the fellow eye (FE) was presented with a (1-0.75)=0.25L decrement and increment on the corresponding sides. Here L is the maximum presentable luminance increment/decrement. A patient with an R of 0.75 would be equally likely to say ghosts on either side were lighter under these conditions – i.e. the ghost presented to the AE was much lighter than the ghost presented to the FE. (B) A demonstration of the task. Viewing the figure through red-green anaglyph glasses the column where the ghosts appear to be most similar in lightness indicates one’s own R. (C) Two examples of VacMan task runs.

Supplementary Fig. 2. Infographic providing a visual summary for study participants and their parents/caregivers, developed with the NIHR Young People’s Advisory Group for eye and vision research (eye-YPAG).

Supplementary Fig. 3. Consort flowchart. In the control group, only one parent/carer opted for atropine treatment, the remaining 15 for occlusion treatment. Observer unmasking occurred once, in the occlusion group.

Supplementary Table 1. Definitions and participant eligibility criteria.

Supplementary Table 2. Reasons for non-enrolment.

Supplementary Table 3: Mean (SEM) values for the secondary outcomes, including Best-Corrected Visual Acuity BCVA) in logMAR, Interocular balance (converted Sbisa values), and Frisby stereoacuity (in seconds of arc), VacMan stereoacuity (in seconds of arc).
