# Supplementary figures and images for: Feasibility of a new ‘balanced binocular viewing’ treatment for unilateral amblyopia in children aged 3–8 years (BALANCE): results of a phase 2a randomised controlled feasibility trial
Source: BMJ Open. 2024 Jul 30;14(7):e082472. doi: 10.1136/bmjopen-2023-082472 (PMC11407205; doi:10.1136/bmjopen-2023-082472)

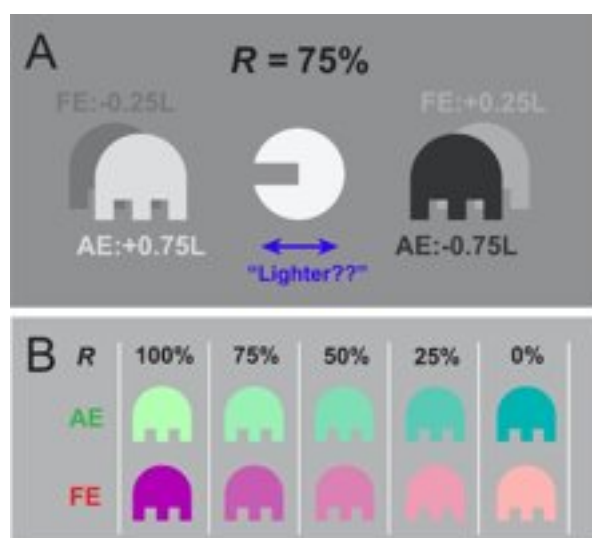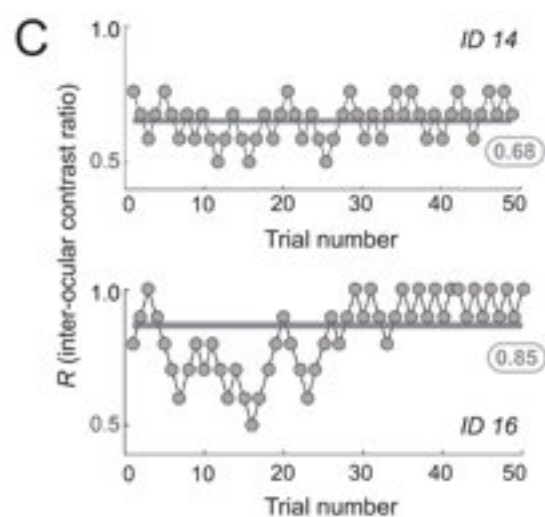

Supplement: online supplemental file 2 [file bmjopen-14-7-s002.pdf]

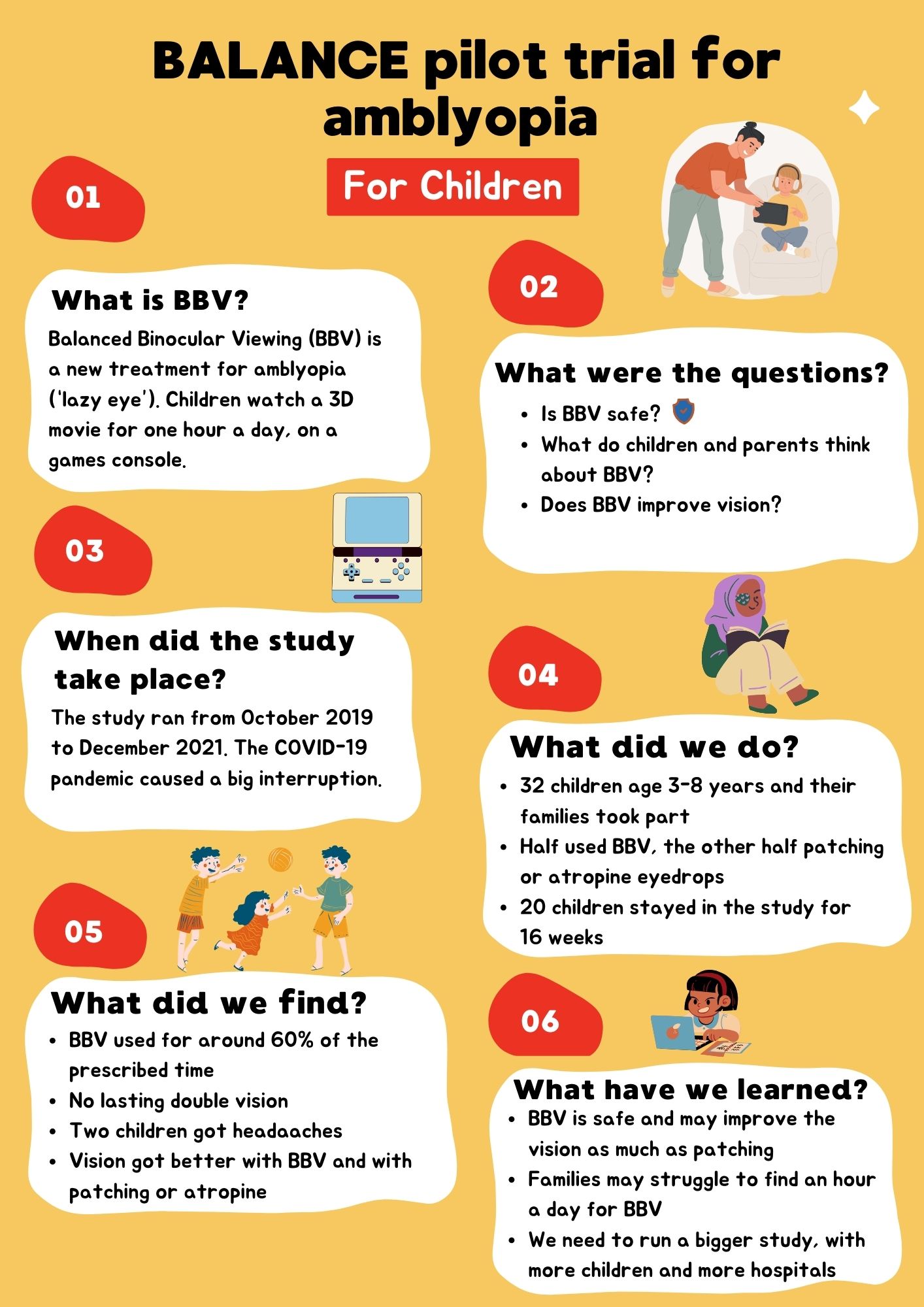

Supplement: online supplemental file 3 [file bmjopen-14-7-s003.jpg]
