# Supplementary material for: Feasibility of a new ‘balanced binocular viewing’ treatment for unilateral amblyopia in children aged 3–8 years (BALANCE): results of a phase 2a randomised controlled feasibility trial
Source: BMJ Open. 2024 Jul 30;14(7):e082472. doi: 10.1136/bmjopen-2023-082472 (PMC11407205; doi:10.1136/bmjopen-2023-082472)
Supplement: online supplemental file 4 [file bmjopen-14-7-s004.pdf]

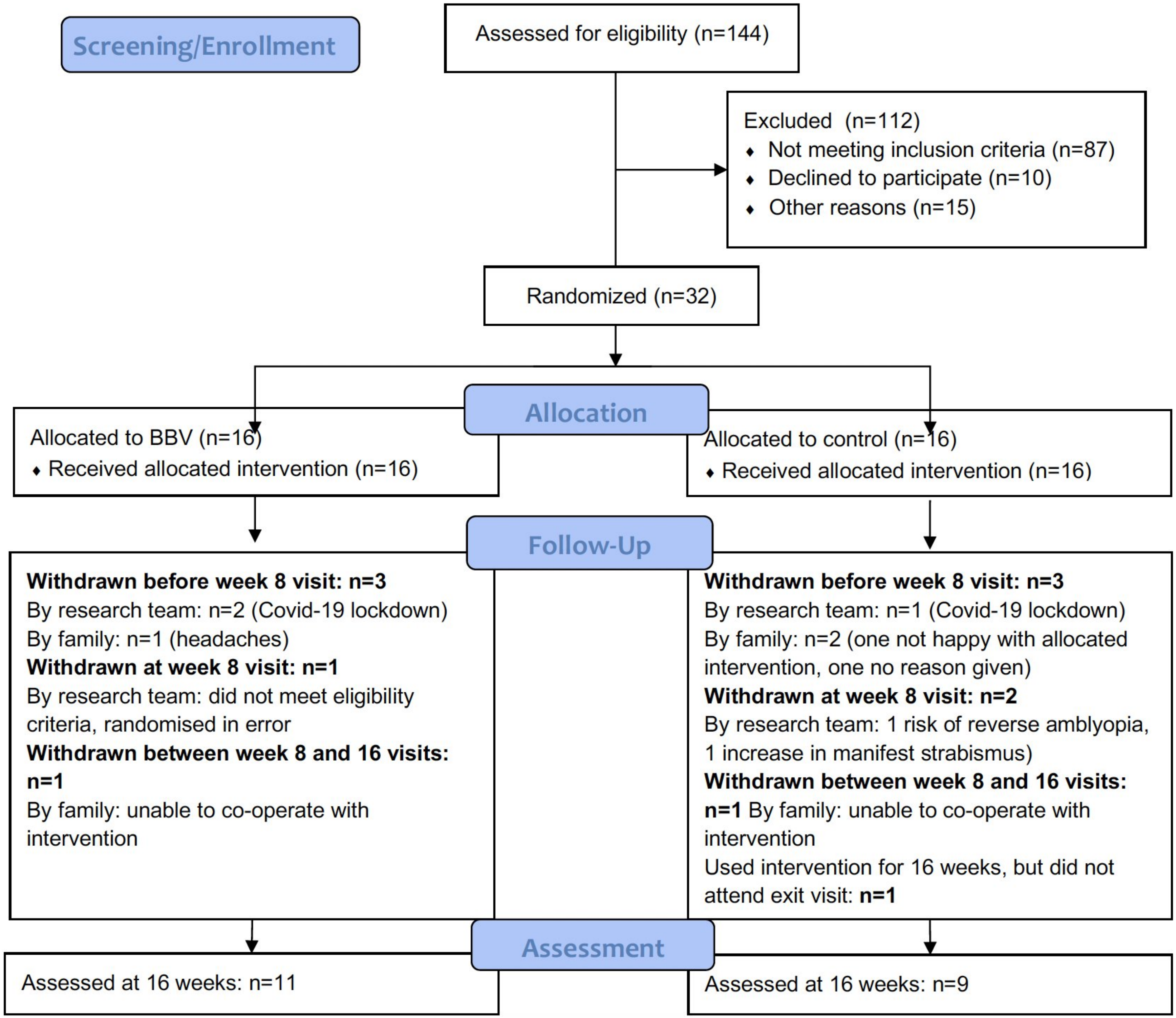
Screening/Enrollment
Assessed for eligibility (n=144)
Excluded (n=112)
Not meeting inclusion criteria (n=87)
Declined to participate (n=10)
Other reasons (n=15)
Randomized (n=32)
Allocation
Allocated to BBV (n=16)
Received allocated intervention (n=16)
Allocated to control (n=16)
Received allocated intervention (n=16)
Follow-Up
Withdrawn before week 8 visit: n=3
By research team: n=2 (Covid-19 lockdown)
By family: n=1 (headaches)
Withdrawn at week 8 visit: n=1
By research team: did not meet eligibility criteria, randomised in error
Withdrawn between week 8 and 16 visits: n=1
By family: unable to co-operate with intervention
Withdrawn before week 8 visit: n=3
By research team: n=1 (Covid-19 lockdown)
By family: n=2 (one not happy with allocated intervention, one no reason given)
Withdrawn at week 8 visit: n=2
By research team: 1 risk of reverse amblyopia, 1 increase in manifest strabismus)
Withdrawn between week 8 and 16 visits: n=1 By family: unable to co-operate with intervention
Used intervention for 16 weeks, but did not attend exit visit: n=1
Assessment
Assessed at 16 weeks: n=11
Assessed at 16 weeks: n=9
