# Supplementary material for: Feasibility of a new ‘balanced binocular viewing’ treatment for unilateral amblyopia in children aged 3–8 years (BALANCE): results of a phase 2a randomised controlled feasibility trial
Source: BMJ Open. 2024 Jul 30;14(7):e082472. doi: 10.1136/bmjopen-2023-082472 (PMC11407205; doi:10.1136/bmjopen-2023-082472)
Supplement: online supplemental file 5 [file bmjopen-14-7-s005.pdf]

**Definitions**<br>Strabismic amblyopia: amblyopia in the presence of esotropia (in-turn squint) with near deviation up to 10 prism dioptres, with or without previous surgical correction, and no significant refractive error (i.e. hypermetropia of less than 1.50 dioptre sphere (DS); American Academy of Ophthalmology, http://www.aao.org/pediatric-center-detail/types-of-amblyopia).<br><br>Refractive/anisometropic amblyopia: amblyopia in the presence of anisometropia (difference in glasses prescription between the two eyes) of ≥0.5 DS of spherical equivalent or ≥1.50 dioptre cylinder (DC) of difference in astigmatism in any meridian which persists after optical adaptation, with no measurable heterotropia (manifest misalignment) at distance or near fixation.<br><br>Combined mechanism amblyopia: amblyopia in the presence of either an esotropia at distance and/or near fixation or a history of strabismus surgery, as well as anisometropia of ≥1.0 DS of spherical equivalent or ≥1.50 DC of difference in astigmatism in any meridian, which persists after optical adaptation.
**Participant eligibility criteria**<br>*Inclusion Criteria*<br>• Age between 3 and 8 years (inclusive)<br>• Unilateral anisometropic, strabismic or combined mechanism amblyopia (see definitions above)<br>• Best-corrected visual acuity (BCVA) in the amblyopic eye worse than 0.20 logMAR, with a difference between the eyes of 0.20 logMAR or more<br>• Completion of optical treatment prior to inclusion in the trial, if applicable<br>• No prior treatment (including occlusion or atropine), other than optical treatment<br><br>*Exclusion Criteria:*<br>• Ocular cause for reduced visual acuity other than amblyopia<br>• Inability/unwillingness to cooperate with the assessment tests<br>• Developmental disorders, learning or neurological disabilities likely to impact adherence to treatment<br>• Photosensitive epilepsy<br>• Prior intraocular surgery<br>• Myopia with spherical equivalent of greater than -6.0 DS<br>• Manifest strabismus greater than 10 prism dioptre with distance or near fixation

Supplementary Table 1: Definitions and participant eligibility criteria.
