# Supplementary material for: Feasibility of a new ‘balanced binocular viewing’ treatment for unilateral amblyopia in children aged 3–8 years (BALANCE): results of a phase 2a randomised controlled feasibility trial
Source: BMJ Open. 2024 Jul 30;14(7):e082472. doi: 10.1136/bmjopen-2023-082472 (PMC11407205; doi:10.1136/bmjopen-2023-082472)
Supplement: online supplemental file 6 [file bmjopen-14-7-s006.pdf]

Reasons for non-enrolment	n
**Not eligible:**	
no longer eligible, as interocular acuity difference improved to less than 0.2 logMAR during optical adaptation	55
Strabismus greater than 10 prism dioptres	7
ineligible, not meeting criteria	10
Not only amblyopia: Abnormal fundus findings, or no amblyopic risk factor identified	2
Occlusion treatment started instead of research appointment	3
Occlusion treatment started whilst study suspended during COVID-19 lockdown	8
Child not able to co-operate (autism spectrum disorder, anxiety)	2
**Declined to take part:**	
Parent/carer did not wish to take part (COVID-19 concerns)	3
Parent/carer did not wish randomisation to new treatment	3
Parent/carer did not wish to take part (personal circumstances: language barrier, CIN plan)	1
Parent/carer did not wish to take part (reason not specified)	1
Study site too far from family's home	1
Family moved out of area	1
**Other:**	
Reason unknown	7
Family did not attend several clinical appointments	7
Impending COVID-19 lockdown and suspension of trial	1
**Total**	112

Supplementary table 2. Reasons for non-enrolment.
