# Supplementary material for: Feasibility of a new ‘balanced binocular viewing’ treatment for unilateral amblyopia in children aged 3–8 years (BALANCE): results of a phase 2a randomised controlled feasibility trial
Source: BMJ Open. 2024 Jul 30;14(7):e082472. doi: 10.1136/bmjopen-2023-082472 (PMC11407205; doi:10.1136/bmjopen-2023-082472)
Supplement: online supplemental file 7 [file bmjopen-14-7-s007.pdf]

| | Occlusion | | BBV | | Treatment effect | |
|---|---|---|---|---|---|---|
| | Baseline | Week 16 | Baseline | Week 16 | Estimate (95% CI) | p-value |
| BCVA | 0.46 (0.05) | 0.26 (0.05) | 0.50 (0.07) | 0.32 (0.08) | 0.03 (-0.11 to 0.16) | 0.67 |
| Suppression (converted Sbisa) | 84.5 (10.85) | 91.3 (4.35) | 85.2 (9.60) | 85.1 (5.11) | -6.9 (-22.6 to 8.73) | 0.36 |
| Stereoacuity (Frisby) | 365.7 (66.9) | 177.9 (45.0) | 155.8 (40.6) | 103.3 (13.9) | 16.5 (-77.7 to 110.8) | 0.70 |
| Stereoacuity (VacMan) | 600.0 (0.0) | 346.5 (229.5) | 479.7 (120.3) | 248.0 (68.1) | - | - |

Supplementary Table 3: Mean (SEM) values for the secondary outcomes, including Best-Corrected Visual Acuity BCVA) in logMAR, Interocular balance (converted Sbisa values), and Frisby stereoacuity (in seconds of arc), VacMan stereoacuity (in seconds of arc).
