# Supplementary material for: Feasibility of a new ‘balanced binocular viewing’ treatment for unilateral amblyopia in children aged 3–8 years (BALANCE): results of a phase 2a randomised controlled feasibility trial
Source: BMJ Open. 2024 Jul 30;14(7):e082472. doi: 10.1136/bmjopen-2023-082472 (PMC11407205; doi:10.1136/bmjopen-2023-082472)
Supplement: Supplementary file 8 [file bmjopen-2023-082472.reviewer_comments.pdf]

PEER REVIEW HISTORY

BMJ Open publishes all reviews undertaken for accepted manuscripts. Reviewers are asked to complete a checklist review form (**http://bmjopen.bmj.com/site/about/resources/checklist.pdf**) and are provided with free text boxes to elaborate on their assessment. These free text comments are reproduced below.

ARTICLE DETAILS

**TITLE (PROVISIONAL)**	Feasibility of a new ‘balanced binocular viewing’ treatment for unilateral amblyopia in children age 3–8 years (BALANCE): results of a phase 2a randomised controlled feasibility trial
**AUTHORS**	Dahlmann-Noor, Annegret; Greenwood, John; Skilton, Andrew; Baker, Daniel; Abbas, Mohamed; Clay, Emma; Khandelwal, Payal; Dunham, Denise; Ludden, Siobhan; Davis, Amanda; Dehbi, Hakim-Moulay; Dakin, Steven C.

VERSION 1 – REVIEW

**REVIEWER**	MiYoung Kwon<br>Northeastern University - Boston Campus
**REVIEW RETURNED**	19-Dec-2023

**GENERAL COMMENTS**	A team of experienced researchers thoroughly conducted an observer-masked phase 2a RCT trial to evaluate the safety, adherence, acceptability, and efficacy of dichoptic therapy (i.e., BBV) as a treatment for amblyopia in young children. Despite the relatively small number of subjects enrolled in the study, its outcomes are informative, providing both quantitative and qualitative measures of safety, adherence and acceptability of dichoptic therapy compared to conventional occlusion therapy. Undoubtedly, this study will capture the great interest of clinicians and vision scientists in the field of amblyopia research.<br><br>This paper is well written and structured, effectively outlining all the outcome measures. The authors made it clear that BBV can serve as a viable alternative therapy, demonstrating comparable functional outcomes without notable adverse effects. I have just a few minor comments that require further clarification and justification:<br><br>1. Although the authors assert favorable outcomes in BCVA and stereoacuity for both dichoptic and occlusion therapy, the absence of a no-treatment group raises uncertainty about whether the observed functional improvements are solely attributed to the treatments. It would be beneficial for the authors to address the absence of a no-treatment group in their study.<br><br>2. From Fig 2, it is evident that six out of ten subjects in the BBV group at baseline exhibit significant suppression of the fellow eye, indicated by an interocular balance point of 0 or 0.3. Also, the average interocular balance for the BBV group stands at 0.5, suggesting balanced vision. Interestingly, both dichoptic and occlusion treatments lead to a decrease in the balance point, indicating apparent suppression of the fellow eye following the treatments.<br>The authors might want to provide insights or comments on these

	observations.<br><br>3. Consideringly the relatively high recurrence rate observed for occlusion therapy, a future study could benefit from a longitude design to evaluate and compare the long-term efficacy of both treatments.

**REVIEWER**	Jiawei Zhou<br>Wenzhou Medical University
**REVIEW RETURNED**	02-Apr-2024

**GENERAL COMMENTS**	Dichoptic training has recently been incorporated into Preferred Practice Patterns (PPPs) guidelines, attracting considerable attention in the field. However, several uncertainties remain, limiting its clinical application. This study conducted a comparative clinical trial of dichoptic training versus conventional occlusion therapy in children aged 3 to 8 with amblyopia. Overall, this represents an important contribution to the field of amblyopia treatment. Regrettably, the study did not meet its recruitment targets, enrolling significantly fewer patients than planned. Consequently, the findings may not adequately address the authors' research questions. Additionally, there are several concerns that I hope the authors will consider addressing:<br><br>The authors designated interocular balance as the primary outcome, using it as a safety indicator. I would appreciate if the authors could provide a thorough explanation in both the Introduction and Discussion sections. Why is interocular balance considered a safety indicator? Previous research suggests that the visual impairment in amblyopia is associated with interocular suppression. The treatment should aim to normalize the abnormal interocular suppression observed in amblyopic patients (abnormal R values at baseline in this study) to the level of normal individuals (is the R value supposed to be 0.5 in your tests for a normal population?). In the discussion, it is mentioned that it is conventionally thought that a reduction in suppression precedes the onset of double vision. However, the measurement and definition of suppression in amblyopia might differ across studies. Is the suppression measurement method used in this study related to the onset of double vision?<br><br>Sample size calculation. The authors mentioned that the target sample size was calculated based on the visual acuity endpoint, yet the primary outcome was the change in interocular balance/suppression at 16 weeks relative to baseline. If the calculation was based on interocular suppression, would the sample size still be appropriate? Furthermore, with only 20 patients completing the 16-week follow-up, which is less than half of the planned sample size (n = 44), there may be concerns regarding statistical power.<br><br>Since interocular balance/suppression is the primary outcome, would it have been more appropriate to allocate participants based on their baseline levels of interocular balance/suppression? There are differences in the baseline levels of interocular balance/suppression between the two groups. Could this impact the results?<br><br>The authors mentioned that to maintain masking in the atropine group, families were asked to discontinue treatment two weeks prior to the study visits at 8 and 16 weeks from baseline. Could this have

	influenced the treatment outcomes, and would it have been more appropriate to continue with occlusion during this period? I suggest the authors add a discussion regarding this aspect.

VERSION 1 – AUTHOR RESPONSE

Reviewer 1
We thank reviewer 1 for the positive and constructive feedback. Below our replies to the specific comments.

1. Although the authors assert favorable outcomes in BCVA and stereoacuity for both dichoptic and occlusion therapy, the absence of a no-treatment group raises uncertainty about whether the observed functional improvements are solely attributed to the treatments. It would be beneficial for the authors to address the absence of a no-treatment group in their study.

In our discussion with families about study design, a no-treatment group was not considered as a viable option, as it was felt that no-treatment, or delayed-treatment, is unethical. In the UK, a pivotal trial in 2003 established the superiority of treatment to no-treatment for unilateral amblyopia (Clarke MP, Wright CM, Hrisos S, Anderson JD, Henderson J, Richardson SR. Randomised controlled trial of treatment of unilateral visual impairment detected at preschool vision screening. BMJ. 2003 Nov 29;327(7426):1251. doi: 10.1136/bmj.327.7426.1251. PMID: 14644966; PMCID: PMC286242), and since then no amblyopia treatment trial has included a no-treatment group.
The aim of amblyopia treatment trials now is to compare novel treatments with gold-standard treatment, i.e. occlusion. We have added this information to the manuscript.

2. From Fig 2, it is evident that six out of ten subjects in the BBV group at baseline exhibit significant suppression of the fellow eye, indicated by an interocular balance point of 0 or 0.3. Also, the average interocular balance for the BBV group stands at 0.5, suggesting balanced vision. Interestingly, both dichoptic and occlusion treatments lead to a decrease in the balance point, indicating apparent suppression of the fellow eye following the treatments.
The authors might want to provide insights or comments on these observations.

The reviewer is correct to point out that the VacMan suppression values at baseline were quite variable, particularly in the BBV group. We do not think, however, that this compromises the conclusions of the study, given for instance that suppression values obtained using Sbisa filters showed a similar lack of change over the course of treatment, as well as no difference between the treatment arms at week 16. We have added text to the discussion to acknowledge this point.

We agree also that it is interesting that the mean suppression values are below 0.5 for both treatments at week 16. Although this may indicate some suppression of the fellow eye, the values do not differ significantly from a mean of 0.5 either for the occlusion group, $t(8)= -1.246$, $p= 0.248$, nor for BBV, $t(9)= -1.604$, $p= 0.143$. This may be a consequence of our small sample size of course, but even if significant this would be a small difference.

3. Considering the relatively high recurrence rate observed for occlusion therapy, a future study could benefit from a longitude design to evaluate and compare the long-term efficacy of both treatments.

We agree with the reviewer, and have added this comment to the Discussion. However, a limiting factor would be the duration of the required observation time – at our institution, median treatment time for amblyopia is 2 years, and the post-treatment observation period would add a further 6 months. It may be difficult to attract funding for a lengthy trial when the “industry standard” is to report outcomes after 3 or 4 months of treatment.

Reviewer 2
We thank reviewer 2 for their helpful comments. We agree that a larger sample size would have been desirable, but as this was a pilot trial, we would argue that the essential questions have still been answered.

The authors designated interocular balance as the primary outcome, using it as a safety indicator. I would appreciate if the authors could provide a thorough explanation in both the Introduction and Discussion sections. Why is interocular balance considered a safety indicator? Previous research suggests that the visual impairment in amblyopia is associated with interocular suppression. The treatment should aim to normalize the abnormal interocular suppression observed in amblyopic patients (abnormal R values at baseline in this study) to the level of normal individuals (is the R value supposed to be 0.5 in your tests for a normal population?). In the discussion, it is mentioned that it is conventionally thought that a reduction in suppression precedes the onset of double vision. However, the measurement and definition of suppression in amblyopia might differ across studies. Is the suppression measurement method used in this study related to the onset of double vision?

When designing this study, we faced the difficulty of satisfying regulatory authorities of the safety of the dichoptic treatment approach. There was particular concern surrounding the question of whether reducing suppression would lead to intractable diplopia in those people with amblyopia who also have strabismus. We reviewed the literature and argued that all cases of intractable diplopia had occurred in adults who had undergone strabismus surgery in childhood. However, we were only allowed to proceed with the trial with a strong emphasis on measuring suppression as safety outcome.
Our previous work indicates that with our dichoptic treatment, visual acuity in the amblyopic eye improves "without breaking suppression", and our group had developed a highly sensitive test to monitor suppression/interocular balance, the VacMan test. The R value in a typical population would indeed be 0.5.
We decided to use this test as the primary outcome, as it indicates both physiological changes with treatment, and satisfied the calls for a safety outcome that in the published literature is linked to the development of diplopia. However, this measurement method has not previously been linked to an onset of double vision vision.
We have added a sentence about this background to the manuscript.

Sample size calculation. The authors mentioned that the target sample size was calculated based on the visual acuity endpoint, yet the primary outcome was the change in interocular balance/suppression at 16 weeks relative to baseline. If the calculation was based on interocular suppression, would the sample size still be appropriate? Furthermore, with only 20 patients completing the 16-week follow-up, which is less than half of the planned sample size (n = 44), there may be concerns regarding statistical power.

To clarify, this trial was a feasibility trial. As indicated in the Manuscript, the primary objective was to evaluate the safety and feasibility of BBV. This is why in our Results we report the feasibility outcomes first. This being said, one does not "power" (i.e. calculate a sample size) for a study based on qualitative feasibility aspects. Given that we envisage visual acuity to be the primary efficacy endpoint of a future large confirmatory phase III trial, it made sense to provide an indication of the power of this relatively small feasibility trial with respect to BCVA. However, from a mechanistic perspective, the interocular balance/suppression is on the pathway to improved visual acuity, which is why in this small feasibility study we preferred to position the VacMan test as our "primary" efficacy endpoint.
At the time of study design, there was not enough data to perform a power/sample size calculation for the VacMan suppression. In this trial, we found that there is no difference in terms of VacMan evolution over time between arms (point estimate: -0.02 (95% CI: -0.28 to 0.23, p=0.87).

Consequently, we do not have a reasonable estimate of treatment effect to perform a retrospective sample size calculation.

Indeed we have pre-post data for 20 out of the 44 patients. Again, our primary objective was not to demonstrate beyond reasonable doubt that BBV would outperform occlusion in terms of visual acuity. “Power” is a design consideration. At analysis stage, power becomes less relevant and it becomes a question of estimation (of treatment effect and associated uncertainty). If the standard deviation of the change had been 0.22 and BBV provided an improvement of 0.225 logMAR above and beyond the change provided by occlusion then with 44 patients we would have had 90% power. Our estimate of the treatment effect is 0.03 logMAR (95% CI: -0.11 to 0.16). This means that our result is inconclusive with respect to BCVA (“absence of evidence isn’t evidence of absence”) and we do know whether there is or isn’t a treatment effect and what its magnitude might be. However, with this study we have gathered data with respect to standard deviation and correlation coefficient between baseline and 16 weeks of BCVA in order to help us refine the sample size calculation for the future trial.

Since interocular balance/suppression is the primary outcome, would it have been more appropriate to allocate participants based on their baseline levels of interocular balance/suppression? There are differences in the baseline levels of interocular balance/suppression between the two groups. Could this impact the results?

The conventional grouping of participants in amblyopia treatment trials is by level of severity of amblyopia. In future trials, we agree with Reviewer 2 that it would be interesting to group participants by baseline interocular balance.
Our study reports the change in interocular balance from baseline, independent from the change in visual acuity, so a different categorisation would not have impacted on our report.

We could have stratified the randomisation based on baseline levels of interocular balance/suppression. This would have reduced the difference at baseline in VacMan between the 2 arms. However, in terms of analysis, given that we are using ANCOVA that adjust for baseline values the difference between the arms at baseline does not impact on the estimated treatment effect (ie difference between BBV and occlusion in the change in BCVA); see also doi: 10.1136/bmj.323.7321.1123.

The authors mentioned that to maintain masking in the atropine group, families were asked to discontinue treatment two weeks prior to the study visits at 8 and 16 weeks from baseline. Could this have influenced the treatment outcomes, and would it have been more appropriate to continue with occlusion during this period? I suggest the authors add a discussion regarding this aspect.

Thank you, we have added a comment to the discussion: “When we designed the present study, parents/carers were given a choice of occlusion or atropine as first-line treatments. This changed during the COVID-19 lockdowns, when monitoring for potential “reverse amblyopia” with atropine was not possible; since then, occlusion treatment has become first-line treatment. In future trials, standard-of-care should be occlusion treatment, which would also reduce the risks linked with non-treatment for two weeks before study visit to maintain masking of atropine treatment.”

VERSION 2 – REVIEW

**REVIEWER**	MiYoung Kwon<br>Northeastern University - Boston Campus
**REVIEW RETURNED**	30-May-2024

**GENERAL COMMENTS**	The revised manuscript is a significant improvement, and the authors have fully addressed my previous concerns and comments.

**REVIEWER**	Jiawei Zhou<br>Wenzhou Medical University
**REVIEW RETURNED**	12-Jun-2024

**GENERAL COMMENTS**	The authors have addressed most of my concerns. However, I am still confused by the logic of the paper. As the authors explain, the main purpose of the study was to test the safety of BBV. I agree that it is important to test the safety of the treatment. I also agree that good amblyopia treatment should try to avoid diplopia. However, the authors try to defend the idea that a treatment that can change binocular balance may be bad because it could lead to diplopia. I do not believe that this argument is adequately supported and the authors do not provide enough a priori data or supporting citations. Is interocular suppression or balance really synonymous with diplopia? One might assume that the interocular balance of normal individuals is quite different from that of the patients in this study; does this mean that all normal individuals suffer from diplopia? Does a more balanced vision in amblyopic patients imply better binocular integration rather than diplopia? I suggest that the authors provide sufficient evidence that "treatment that alters interocular balance causes diplopia." If not, the authors should revise their statement.

**REVIEWER**	Jiawei Zhou<br>Wenzhou Medical University
**REVIEW RETURNED**	12-Jun-2024

**GENERAL COMMENTS**	The authors have addressed most of my concerns. However, I am still confused by the logic of the paper. As the authors explain, the main purpose of the study was to test the safety of BBV. I agree that it is important to test the safety of the treatment. I also agree that good amblyopia treatment should try to avoid diplopia. However, the authors try to defend the idea that a treatment that can change binocular balance may be bad because it could lead to diplopia. I do not believe that this argument is adequately supported and the authors do not provide enough a priori data or supporting citations. Is interocular suppression or balance really synonymous with diplopia? One might assume that the interocular balance of normal individuals is quite different from that of the patients in this study; does this mean that all normal individuals suffer from diplopia? Does a more balanced vision in amblyopic patients imply better binocular integration rather than diplopia? I suggest that the authors provide sufficient evidence that "treatment that alters interocular balance causes diplopia." If not, the authors should revise their statement.

VERSION 2 – AUTHOR RESPONSE

Reviewer: 1

Dr. MiYoung Kwon, Northeastern University - Boston Campus

Comments to the Author:

The revised manuscript is a significant improvement, and the authors have fully addressed my previous concerns and comments.

Thank you.

Reviewer: 2

Prof. Jiawei Zhou, Wenzhou Medical University

Comments to the Author:

The authors have addressed most of my concerns. However, I am still confused by the logic of the paper. As the authors explain, the main purpose of the study was to test the safety of BBV. I agree that it is important to test the safety of the treatment. I also agree that good amblyopia treatment should try to avoid diplopia. However, the authors try to defend the idea that a treatment that can change binocular balance may be bad because it could lead to diplopia. I do not believe that this argument is adequately supported and the authors do not provide enough a priori data or supporting citations. Is interocular suppression or balance really synonymous with diplopia? One might assume that the interocular balance of normal individuals is quite different from that of the patients in this study; does this mean that all normal individuals suffer from diplopia? Does a more balanced vision in amblyopic patients imply better binocular integration rather than diplopia? I suggest that the authors provide sufficient evidence that "treatment that alters interocular balance causes diplopia." If not, the authors should revise their statement.

Thank you for requesting further clarification. We have amended the relevant paragraph in the introduction and added relevant references:

The primary objective for this trial was to evaluate the safety of BBV at 16 weeks from randomisation by exploring whether BBV causes a change in suppression/interocular balance, which may precede the development of double vision (diplopia) [1] [2]. Changes to the strength of either binocular fusion or interocular suppression can alter the likelihood of diplopia [3] [4]. Diplopia can occur in normal individuals through variations in either of those factors [3] [4], and amblyopia involves more extreme changes. Intractable, irreversible diplopia has to date never been observed in dichoptic treatment trials, though occasional, intermittent diplopia has been reported [5] [6], both with dichoptic treatment and standard of care [7].

VERSION 3 – REVIEW

**REVIEWER**	Jiawei Zhou<br>Wenzhou Medical University
**REVIEW RETURNED**	08-Jul-2024

**GENERAL COMMENTS**	My suggestions have been addressed and the manuscript is strengthened.
